# Prenatal Sonographic Features of CHARGE Syndrome

**DOI:** 10.3390/diagnostics11030415

**Published:** 2021-02-28

**Authors:** Kuntharee Traisrisilp, Wisit Chankhunaphas, Rekwan Sittiwangkul, Chureerat Phokaew, Vorasuk Shotelersuk, Theera Tongsong

**Affiliations:** 1 Department of Obstetrics and Gynecology, Faculty of Medicine, Chiang Mai University, Chiang Mai 50200, Thailand; kuntharee.t@cmu.ac.th (K.T.); wisit.chank@gmail.com (W.C.); 2 Department of Pediatrics, Faculty of Medicine, Chiang Mai University, Chiang Mai 50200, Thailand; rekwan@gmail.com; 3 Center of Excellence for Medical Genomics, Medical Genomics Cluster, Department of Pediatrics, Faculty of Medicine, Chulalongkorn University, Bangkok 10332, Thailand; Chureerat.P@chula.ac.th (C.P.); vorasuk.s@chula.ac.th (V.S.)

**Keywords:** CHARGE syndrome, fetal imaging, prenatal diagnosis, ultrasound

## Abstract

CHARGE syndrome is a rare autosomal dominant disorder, associated with coloboma (C), heart defects (H), choanal atresia (A), retardation of growth and/or central nervous system (R), genitourinary anomalies (G) and ear abnormalities (E). Prenatal diagnosis of the syndrome is very rare but may be suspected when a combination of such abnormalities is identified. We describe a prenatally suspected case of CHARGE syndrome due to unique findings of cardiac defects (DORV) in combination with minor clues, including a structurally malformed ear with persistent non-response to an acoustic stimulation (which has never been prenatally described elsewhere), renal malrotation and growth restriction. Postnatal diagnosis was made based on confirmation of the prenatal findings and additional specific findings of bilateral coloboma, choanal atresia and ear canal stenosis. Finally, molecular genetic testing by whole exome sequencing of the neonate and her parents revealed a novel de novo heterozygous frameshift c.3506_3509dup variant in the *CHD7* gene, confirming the clinical diagnosis of CHARGE syndrome. In conclusion, we describe unique prenatal features of CHARGE syndrome. Educationally, this is one of the rare examples of CHARGE syndrome, comprising all of the six specific anomalies as originally described; it is also supported by the identification of a specific genetic mutation. The identified genetic variant has never been previously reported, thereby expanding the mutational spectrum of *CHD7*. Finally, this case can inspire prenatal sonographers to increase awareness of subtle or minor abnormalities as genetic sonomarkers.

## 1. Introduction

CHARGE syndrome is a rare, autosomal dominant genetic disorder with an incidence of approximately 1 in 10,000 births [1]. The majority of cases (65–70%) are caused by the loss of function pathogenic variants in the *CHD7* gene [2]. CHARGE refers to a disorder associated with ocular coloboma (C), heart defects (H), choanal atresia (A), retardation of growth and/or central nervous system development (R), genito-urinary anomalies (G) and ear abnormalities (E). This syndrome was first reported in 1979 by Hittner et al. [3] and Hall [4]. The term CHARGE was introduced by Pagon et al. [5], in 1981. Conventionally, diagnosis is usually made by clinical criteria. Though there is no consensus, most people refer to the criteria proposed by Blake et al. [6] and further modified by Verloes [7] who highlighted the importance of the 3C major criteria (coloboma, choanal atresia, and hypoplasia of the semicircular ear canals). Other minor criteria include rhombencephalic dysfunction (brainstem and cranial nerves III to XII, including sensorineural deafness) malformation of the ear, malformation of the mediastinum (heart, esophagus) and mental retardation. Verloes [7] proposed the criteria for typical CHARGE: three majors (3C triad) OR two majors plus two minors; partial CHARGE: two majors plus one minor; and atypical CHARGE: two majors but no minors OR one major plus two minors. After identification of mutations in the *CHD7* gene resulting in several phenotypic abnormalities, Hale et al. [8] recently proposed the inclusion of results of *CHD7* testing in the clinical criteria.

Most cases were diagnosed in neonatal or childhood period. Prenatal diagnosis has been rarely reported [9,10,11,12,13]. The syndrome is usually associated with severe morbidity and may even be life threatening. Therefore, early prenatal diagnosis is critical, either by clinical criteria or molecular genetic testing, because it can be informative for the prognosis, guidance of delivery preparation, postnatal care plan for the parents and even the option of termination of pregnancy, especially in cases of diagnosis in the pre-viable stage. Nevertheless, the number of cases with prenatal diagnosis is very limited. This may be due to the fact that most abnormalities of the syndrome are prenatally subtle or presented as an isolated abnormality and phenotypic diversity. Accordingly, more new prenatal cases are needed to enhance the literature for future analysis, especially on prenatal findings and management. The aim of this study is to describe unique prenatal features of CHARGE syndrome and also to educationally illustrate an intriguing case of CHARGE syndrome, comprising all of the six specific anomalies as originally described; it is also confirmed by the identification of a pathogenic genetic variant in the *CHD7* gene.

## 2. Case Presentation

**Figure 1:**
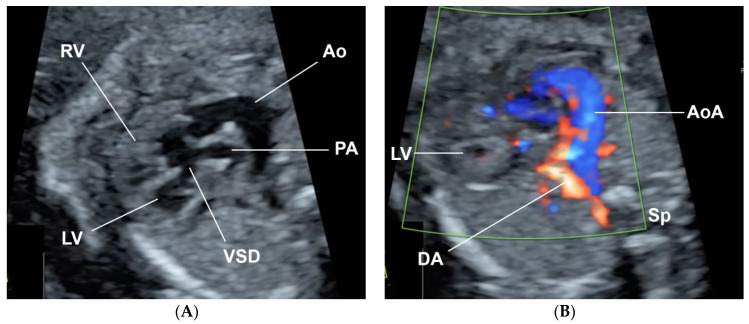
Double-outlet right ventricle (DORV): (**A**) TOF type, small pulmonary artery (PA) and (**B**) reverse flow in the ductus arteriosis (DA) (Ao: ascending aorta; AoA: aortic arch; LV: left ventricle; RV: right ventricle; Sp: spine; VSD: ventricular septal defect).

**Figure 2:**
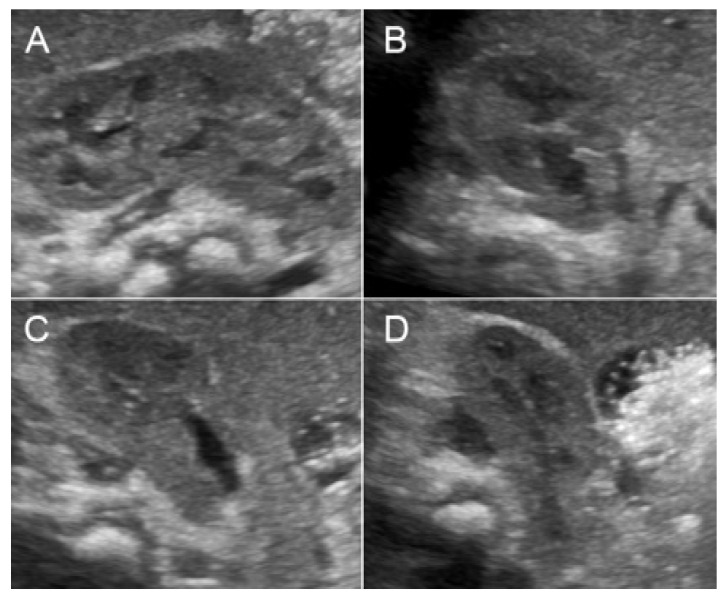
Malrotation of the right kidney with abnormally lateral-posterior renal pelvis (**A**) sagittal scan; (**B**–**D**) cross-sectional scan at upper, mid and lower portion).

**Figure 3:**
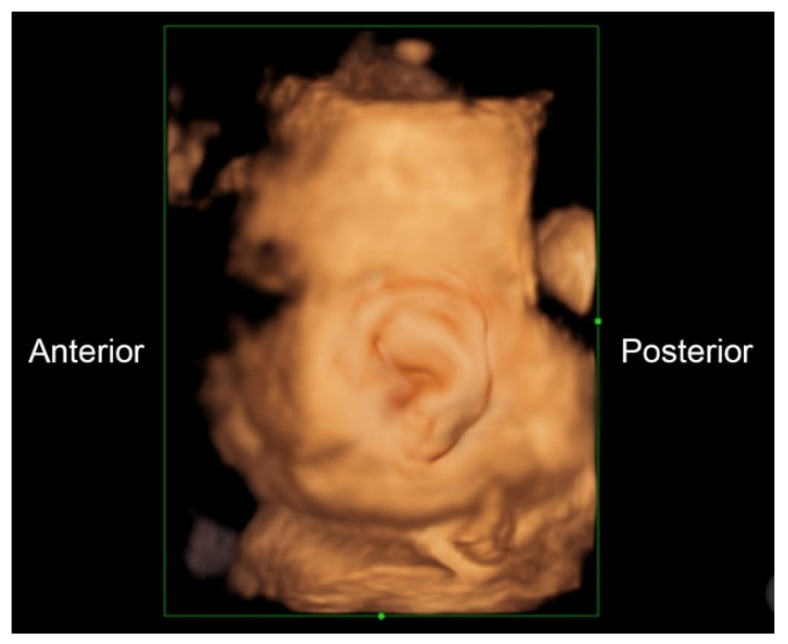
3D-Ultrasound: Abnormal ear; markedly prominent superior crus of anti-helix.

**Figure 4:**
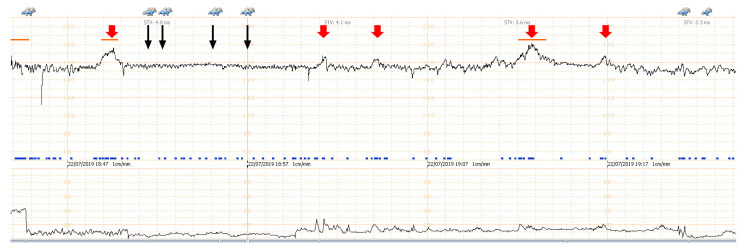
Non-stress test/acoustic stimulation: the fetus at 34 weeks of gestation shows spontaneous fetal heart rate (FHR) acceleration (thick red arrow); but no response to acoustic stimulation (thin black arrow).

A 32-year-old pregnant woman, G4 P2102, attended her first visit of antenatal care at 23^+3^ weeks of gestation. Ultrasound examination for anomaly screening demonstrated cardiac defects, including double-outlet right ventricle: DORV-TOF type, with severe pulmonary stenosis (Figure 1) and left superior vena cava. Detailed ultrasound showed no associated abnormalities. Fetal biometry was consistent with gestational age except abdominal circumference and estimated fetal weight, which were relatively low (at 10th percentile), reflecting some degree of growth restriction. However, detailed ultrasound on the follow-up scans at 28 weeks of gestation showed subtle abnormalities, including malrotation of both kidneys, the hilum or renal pelvis facing posteriorly to the abdominal wall (Figure 2). Furthermore, 3D-ultrasound revealed abnormal external ear structure (markedly prominent crus of anti-helix) (Figure 3). Non-stress tests (NST) showed spontaneous fetal heart rate (FHR) accelerations (normal reactive tests) (23^+3^ weeks). Interestingly, the fetus showed persistent non-response to acoustic stimulation tests at 26, 30, 32, 36 and 38 weeks (no FHR accelerations as well as no quickening perceived by ultrasound) (Figure 4), probably reflective of auditory dysfunction. Based on the findings of heart defect, ear defect, renal defect and growth restriction, several differential diagnoses were listed, including CHARGE syndrome. Theoretically, fetal blood sampling for molecular genetic tests should be performed. Nevertheless, since no lethal condition was identified and the couple wanted to continue pregnancy regardless of investigation results, prenatal invasive diagnosis was avoided, and we waited for postnatal work-up instead. She had no significant underlying disease and no familial history of hereditary diseases. Her pregnancy was uneventful except that she developed gestational diabetes (GDM) at 28 weeks of gestation, which was well-controlled with diabetic diet.

**Figure 5:**
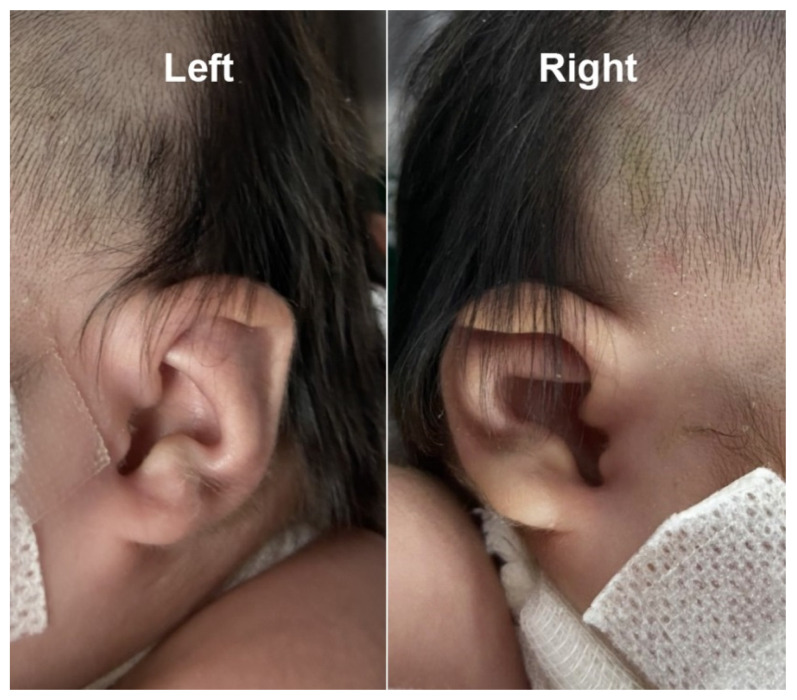
Abnormal gross appearance of the left and right ear; clipped-off helix.

**Figure 6:**
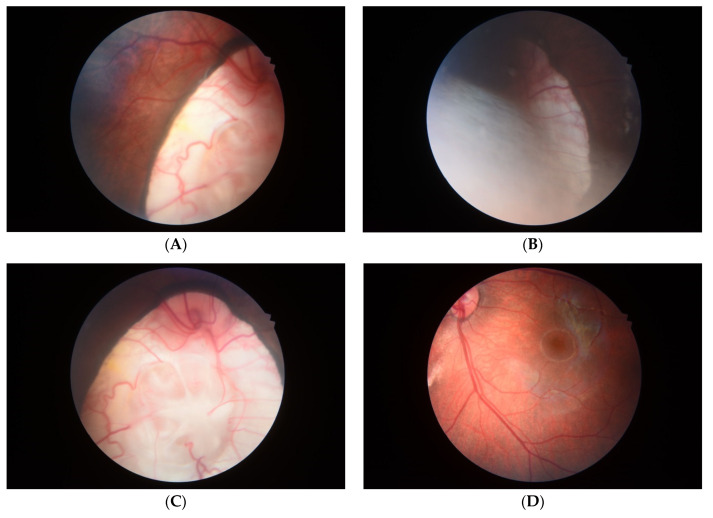
Fundoscopic findings: (**A**,**B**) (Left) Retinochoroidal coloboma: characterized by missing pieces of retinal and choroidal tissue, which can result in a large blind spot; (**C**) (Right) Optic disc coloboma: the optic disc is vertically oval with excavation. The retinal vessels have abnormal origins. (**D**) (Left) Normal optic nerve and macula.

**Figure 7:**
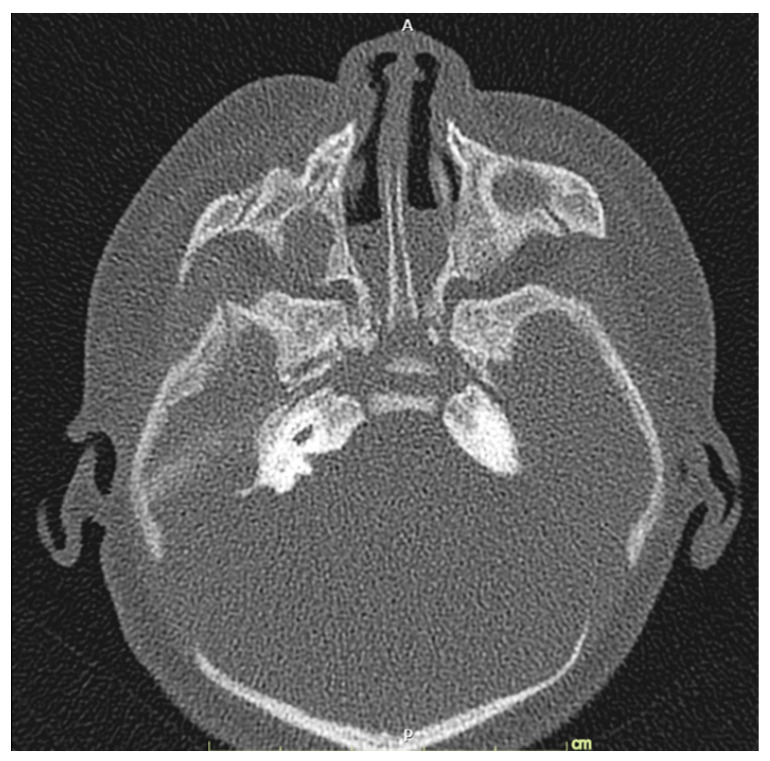
Bilateral bony choanal atresia: narrowing of bilateral posterior choanae, measuring about 0.29 and 0.22 cm on right and left sides, respectively.

At 38^+4^ weeks, she had vaginal delivery, giving birth to a female baby, with birthweight of 2580 g (9th percentile of WHO growth chart); APGAR score 8 and 9 at 1 and 5 min, respectively, with excessive secretion in both nostrils. The left ear showed a clipped off helix (Figure 5). Otoscopic examination showed bilateral ear canal stenosis with cerumen. Funduscopic examination revealed optic disc coloboma on the right eye and choroidal coloboma on the left eye (Figure 6). Abdominal ultrasound revealed mal-rotation of both kidneys but with normal size and echogenicity of the renal parenchyma; no dilatation of the pelvicalyceal system was observed. Neonatal echocardiography confirmed the prenatal findings. Because of inability to pass nasogastric tube, bilateral choanal atresia was suspected and confirmed by CT scans of the head (Figure 7).

**Figure 8:**
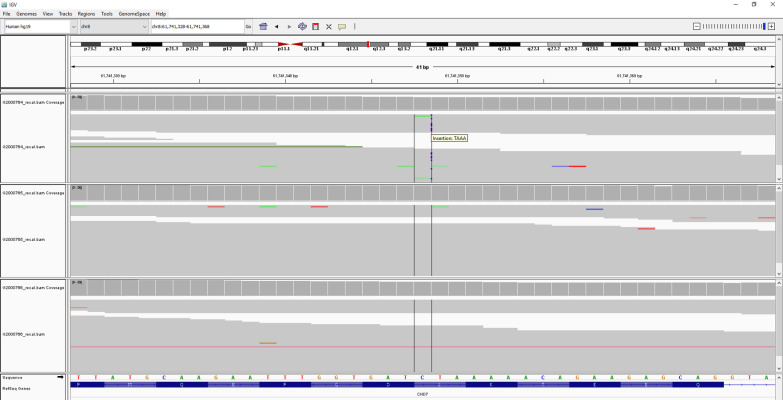
Trio WES: patient’s nucleotides sequence showed nucleotides insertion (C > CTAAA, blue dot in top panel) when mapping with her parents for reference (two bottom panels) causing frameshift c.3506_3509dup variant.

Prenatally, this case was suspected of CHARGE syndrome based on the findings of cardiac defects, ear abnormalities, growth restriction and renal abnormality. The diagnosis was postnatally confirmed by additional specific findings of coloboma, choanal atresia and ear canal stenosis. Finally, the diagnosis was confirmed by molecular genetic testing, as follows:

Molecular genetic study: Trio WES-analysis was performed for the neonate and her parents. Genomic DNA was isolated from peripheral blood leukocytes. The DNA samples were enriched by SureSelect Human All Exon V7 kit, Agilent: Santa Clara, CA, USA and sequenced onto Illumina HiSeq 4000 Sequencer Illumina: San Diego, CA, USA. A novel de novo heterozygous frameshift c.3506_3509dup variant (chr8:61741348 C > CTAAA, p.K1170Nfs*39) in the *CHD7* gene was identified in the neonate (Figure 8), but not her parents. This variant has not been identified in the ExAc, Gnomad, Clinvar and the in-house 3206 Thai Exome databases.

The baby underwent a left modified Blalock–Taussig shunt (MBTS) as palliative surgery at one month of age, with a successful outcome resulting in a well-oxygenated condition (oxygen saturation 98–100%). At the time of writing this report, the definitive corrective surgery has not been performed. The neonate was taken care of and followed-up by ophthalmologists and ENT specialists (for auditory brainstem response test).

## 3. Discussion

Lessons gained from this study are as follows: (1) CHARGE syndrome may have some sonographic clues that enhance the possibility of prenatal diagnosis. Though several abnormalities of the syndrome are difficult to detect by routine prenatal ultrasound, with careful examinations, subtle anomalies may be disclosed. For example, in our case, at first only cardiac defect was appreciated, but minor findings, such as ear abnormalities and kidney mal-rotation, were visualized on follow-up scans. Therefore, in case of an isolated anomaly at the first scan, we emphasize follow-up scans to disclose minor late-appearing sonomarkers to enhance pattern recognition. (2) To the best of our knowledge, this is the first report on the detection of fetal auditory dysfunction using simple tools in daily practice to support the diagnosis of CHARGE syndrome. (3) Educationally, the case presented here is one of the rare and intriguing examples of CHARGE syndrome, comprising all of the six specific anomalies as originally described; it is also supported by the most solid evidence of a de novo heterozygous frameshift mutation in the *CHD7* gene. Accordingly, the diagnosis in this case is absolutely certain.

Prenatal diagnosis of CHARGE syndrome is critical but rarely made. This is due to the fact that prenatal ultrasound has some limitations in diagnosis of the syndrome since it has a wide spectrum of abnormalities and most of them, especially the primary specific defects, such as coloboma, choanal atresia and auditory deficit, cannot be visualized by prenatal ultrasound. Nevertheless, several minor criteria may be first appreciated, such as DORV in this case, leading to further disclosure of the subtle abnormalities with more careful examination or serial scans for late-appearing markers, fetal MRI in some selected cases, especially in cases suspected of CNS abnormalities [14], or prenatal genetic testing when the pattern is mixed-up. Pattern recognition of the syndrome is useful as seen in this case. Nevertheless, the patterns of anomalies of several syndromes largely overlap, which must be taken into account in differential diagnosis; for example, isolated congenital heart defects, Kabuki syndrome (typical facial gestalt, postnatal growth deficiency, congenital heart defects, hearing loss and intellectual disability, skeletal, dermatoglyphic, genitourinary, and ophthalmologic anomalies, including coloboma), VACTREL association (Vertebral abnormalities, anal atresia, cardiac, renal/adial ray, esophageal and limb defects), Smith-Lemli-Opitz syndrome (IUGR, genital and cardiac anomalies), Joubert spectrum (ventriculomegaly, polydactyly, renal abnormalities, Dandy–Walker variant, cephalocele) and 22q11.2 deletion syndrome [1]. For example, some cases of CHARGE syndrome have thymus agenesis and conotruncal heart defects, highlighting the clinical overlap with a 22q11.2 deletion. In cases of normal 22q11.2, CHARGE syndrome should be highly considered. Then, thorough evaluation of the ears with auditory function, and choana (if possible), as well as other subtle clues (mild ventriculomegaly, thymus hypoplasia, arhinencephaly, etc.) should be performed. Fetal brain MRI may be useful. When the diagnosis is highly suspected, DNA analysis of *CHD7* should be strongly considered.

Ear structural abnormality in our case was first subjectively diagnosed, but it seems to be more significant when combined with its function. As already known, fetuses are very sensitive to acoustic stimulation, routinely used in antenatal surveillance, leading to FHR accelerations and fetal quickening. We took advantage of this stimulation to test the auditory perception. Because the fetus persistently showed no response to acoustic stimulation, either by FHR acceleration or quickening in spite of having spontaneous FHR acceleration and quickening, it may reasonably be concluded that the fetus was likely to have auditory dysfunction, probably associated with structural abnormality of the ear canal and external ear abnormality, as seen on 3D-ultrasound.

Interestingly, DORV, which is rarely described as a first clue, was the first clue in this case, leading to final diagnosis of CHARGE syndrome. DORV could be isolated or a part of several syndromes, especially trisomy 18. In this case, there were no other typical findings commonly seen in trisomy 18, such as abnormal hand posture, cleft lip/palate, omphalocele, etc. Furthermore, this is the first report on bilateral renal mal-rotation (without abnormality of renal structures) as a part of the prenatal features of CHARGE syndrome. This minor disorder could be simply overlooked in daily practice, but this report shows that it may be a genetic sonomarker.

The insertion of the four nucleotides, TAAA, leading to a frameshift has never been previously reported. This expands the mutational spectrum of the *CHD7* gene. Since the genetic variant is de novo, as evidenced by its absence in the leukocytes of the proband’s parents, the recurrence risk for the next child is low.

As mentioned earlier, prenatal diagnosis is very limited. Fetal MRI may be more useful in identification of subtle abnormalities of CNS. However, fetal MRI is not a primary tool for fetal anomaly screening. Therefore, the first clue for prenatal diagnosis is usually initiated by some abnormalities on ultrasound screening. Unfortunately, most anomalies are subtle and difficult to identify by standard ultrasound examination, for example, coloboma and choanal atresia. A literature review indicates that CHARGE syndrome has been reported prenatally only a very limited number of times [9,10,11,12,13,15,16]. Prenatal sonographic findings that may be helpful are summarized in Table 1.

## 4. Conclusions

In conclusion, the case presented here is unique and educational. DORV was the first clue, leading to the detection of associated subtle abnormalities, e.g., ear abnormalities with persistent non-response to acoustic stimulation, renal mal-rotation and growth restriction. Postnatal diagnosis was made based on the confirmation of the prenatal findings and additional specific findings of coloboma, choanal atresia and auditory canal stenosis. Finally, the diagnosis was confirmed by genetic testing (a de novo heterozygous frameshift c.3506_3509dup variant in *CHD7*). Educationally, this is a rare and interesting case of CHARGE syndrome, comprising all of the six specific anomalies as originally described. It is also supported by the identification of a specific genetic mutation. Finally, this case can inspire prenatal sonographers to increase awareness of subtle or minor abnormalities as genetic sonomarkers.

## Figures and Tables

**Table 1:** Prenatal sonographic features of CHARGE syndrome [9,10,11,12,13,15,16], including the findings in the case presented here.

Sonographic Findings	Total; *n*: 19 (%)
Coloboma	0 (0.0%)
Cardiac defects (TOF 2, DORV 1; others 2)	5 (26.3%)
Choanal atresia	1 (5.3%)
Cleft lip	3 (15.8%)
External ear abnomalities	3 (15.8%)
semicircular canal agenesis	1 (5.3%)
CNS anomalies; mild ventriculomegaly (2) abnormal olfactory sulci (2) abnormal choroid plexus (1)	5 (26.3%)
Thymic hypoplasia	1 (5.3%)
Genito-renal anomalies	6 (31.6%)
Limb/skeletal (polydactyly; 1)	1 (5.3%)
Esophageal anomalies	2 (10.5%)
Polyhydramnios	6 (31.6%)
Fetal growth restriction	3 (15.8%)
No response to acoustic stimulation	1 (5.3%)

TOF: tetralogy of Fallot; DORV: double-outlet right ventricle.

## Data Availability

The data of this report are available from the corresponding authors upon request.
